# Imatinib inhibits pericyte-fibroblast transition and inflammation and promotes axon regeneration by blocking the PDGF-BB/PDGFRβ pathway in spinal cord injury

**DOI:** 10.1186/s41232-022-00223-9

**Published:** 2022-09-26

**Authors:** Fei Yao, Yang Luo, Yan-Chang Liu, Yi-Hao Chen, Yi-Teng Li, Xu-Yang Hu, Xing-Yu You, Shui-Sheng Yu, Zi-Yu Li, Lei Chen, Da-Sheng Tian, Mei-Ge Zheng, Li Cheng, Jue-Hua Jing

**Affiliations:** 1 Department of Orthopedics, The Second Affiliated Hospital of Anhui Medical University, Anhui Medical University, Hefei, 230032 Anhui Province China; 2 School of Pharmacy, Anhui Medical University, Hefei, 230032 Anhui Province China

**Keywords:** Spinal cord injury, Pericyte, Transition, Endothelial cell, PDGF, Fibrotic scar

## Abstract

**Background:**

Fibrotic scar formation and inflammation are characteristic pathologies of spinal cord injury (SCI) in the injured core, which has been widely regarded as the main barrier to axonal regeneration resulting in permanent functional recovery failure. Pericytes were shown to be the main source of fibroblasts that form fibrotic scar. However, the mechanism of pericyte-fibroblast transition after SCI remains elusive.

**Methods:**

Fibrotic scarring and microvessels were assessed using immunofluorescence staining after establishing a crush SCI model. To study the process of pericyte-fibroblast transition, we analyzed pericyte marker and fibroblast marker expression using immunofluorescence. The distribution and cellular origin of platelet-derived growth factor (PDGF)-BB were examined with immunofluorescence. Pericyte-fibroblast transition was detected with immunohistochemistry and Western blot assays after PDGF-BB knockdown and blocking PDGF-BB/PDGFRβ signaling in vitro. Intrathecal injection of imatinib was used to selectively inhibit PDGF-BB/PDGFRβ signaling. The Basso mouse scale score and footprint analysis were performed to assess functional recovery. Subsequently, axonal regeneration, fibrotic scarring, fibroblast population, proliferation and apoptosis of PDGFRβ^+^ cells, microvessel leakage, and the inflammatory response were assessed with immunofluorescence.

**Results:**

PDGFRβ^+^ pericytes detached from the blood vessel wall and transitioned into fibroblasts to form fibrotic scar after SCI. PDGF-BB was mainly distributed in the periphery of the injured core, and microvascular endothelial cells were one of the sources of PDGF-BB in the acute phase. Microvascular endothelial cells induced pericyte-fibroblast transition through the PDGF-BB/PDGFRβ signaling pathway in vitro. Pharmacologically blocking the PDGF-BB/PDGFRβ pathway promoted motor function recovery and axonal regeneration and inhibited fibrotic scar formation. After fibrotic scar formation, blocking the PDGFRβ receptor inhibited proliferation and promoted apoptosis of PDGFRβ^+^ cells. Imatinib did not alter pericyte coverage on microvessels, while microvessel leakage and inflammation were significantly decreased after imatinib treatment.

**Conclusions:**

We reveal that the crosstalk between microvascular endothelial cells and pericytes promotes pericyte-fibroblast transition through the PDGF-BB/PDGFRβ signaling pathway. Our finding suggests that blocking the PDGF-BB/PDGFRβ signaling pathway with imatinib contributes to functional recovery, fibrotic scarring, and inflammatory attenuation after SCI and provides a potential target for the treatment of SCI.

**Supplementary Information:**

The online version contains supplementary material available at 10.1186/s41232-022-00223-9.

## Background

Spinal cord injury (SCI) leads to the destruction of the blood-spinal cord barrier (BSCB), triggering a cascade of reactions to fibrotic scar in the injury lesion core [1–3]. Fibrotic scar is composed of fibroblasts and extracellular matrix, which can limit the inflammatory response and promote wound healing [3, 4]. However, the fibrotic scar also forms a strong physical barrier to inhibit the new axon from passing through the injured site, causing persistent motor and sensory dysfunction [5].

In the normal central nervous system, pericytes, also known as mural cells, connect with microvascular endothelial cells, nerve cells, and astrocytes to form a neurovascular unit [6]. Pericytes have important physiological functions, such as maintaining the BSCB, regulating cerebral blood flow, and promoting microvessel maturation [7, 8]. In addition, pericytes have the multidifferentiation potential characteristic of mesenchymal stem cells, which can transdifferentiate into granulocytes, osteoblasts, and adipocytes [9, 10]. In kidney injury, pericytes can transdifferentiate into (myo)fibroblasts to participate in fibrosis [11]. Microvessels show an excessive increase in density and a disordered distribution within the lesion core after SCI [12]. Stromal pericytes detached from the blood vessel wall are the main source of fibroblasts that form fibrotic scar after SCI, accounting for approximately 10% of all PDGFRβ^+^ pericytes [3, 13]. The activated and proliferating stromal pericytes present a fibroblast-like phenotype, secreting extracellular matrix and enveloping macrophages to form a honeycomb structure. Knockout of NG2^+^ pericytes and inhibition of the proliferation of PDGFRβ^+^ type A pericytes could prevent the formation of fibrotic scar [5, 14]. However, the molecular mechanism underlying pericyte-fibroblast transition after SCI is poorly understood.

Platelet-derived growth factor (PDGF) is a cytokine containing five isotypes (PDGF-AA, -BB, -AB, -CC, and -DD) that can activate downstream PDGF receptor *α* or *β* in a paracrine or autocrine manner under physiological or pathological conditions [15]. PDGF-BB/PDGFRβ signaling has been demonstrated to recruit pericytes and promote microvessel maturation during angiogenesis, in which microvascular endothelial cells are the main source of PDGF-BB [16, 17]. In addition, the PDGF-BB/PDGFRβ signaling pathway is widely involved in pericyte-(myo)fibroblast transition in kidney, lung, and cardiac fibrosis [15]. Imatinib, as a selective PDGFRβ inhibitor, has been known to inhibit the fibrosis of systemic sclerosis, idiopathic pulmonary fibrosis, and age-related macular degeneration in multiple clinical trials [18–20]. Considering robust angiogenesis after SCI and the close interaction between microvascular endothelial cells and pericytes, we speculate that microvascular endothelial cells induce pericyte-fibroblast transition to promote fibrotic scar formation through the PDGF-BB/PDGFRβ signaling pathway.

In this study, we found that pericytes detached from the blood vessel wall and transitioned into fibroblasts. Microvascular endothelial cells induced pericyte-fibroblast transition through the PDGF-BB/PDGFRβ signaling pathway. Blocking the PDGF-BB/PDGFRβ signaling pathway could promote functional recovery, attenuate fibrotic scar formation, and reduce microvessel leakage after SCI.

## Methods

### Animals

C57BL/6J female mice were purchased from the Experimental Animal Center of Anhui Medical University and bred in a pathogen-free animal cage. All animal experiments were performed following the Institutional Animal Care guidelines of Anhui Medical University and approved by the Institutional Animal Ethical Committee of Anhui Medical University. Mice were housed in an environment of suitable temperature and humidity with a 12:12-h light-dark cycle and free access to food and water. The sample size was determined according to previous common experiences and was not determined by statistical methods. The litter mice were randomly divided into different groups for subsequent experiments.

### Crush spinal cord injury model

All SCI surgery instruments were performed under anesthesia with intraperitoneal injection of sodium pentobarbital according to our previous method [21]. Female mice aged 8–10 weeks underwent a single laminectomy at the T10 level. Severe spinal cord crush injury was induced by the constant compression of No. 5 Dumont forceps (Fine Science Tools, Foster City, CA) from the space between the pedicles for 5 s. A clear linear red clamp mark was observed at the spinal forceps site. The mice were placed in an incubator (22–24°C) until fully awakened. All mice were given fluid supplementation and analgesia after surgery. Bladder evacuation was performed twice a day to prevent urinary retention.

### Histology and immunofluorescence staining

Animals were anesthetized with an intraperitoneal injection of sodium pentobarbital (50 mg/kg) and transcranially perfused with phosphate-buffered saline (PBS, BL601A, Biosharp, China) followed by 4% paraformaldehyde (PFA, G1101, Servicebio, China). Spinal cord tissues were dissected and postfixed in 4% PFA overnight at 4°C. The tissues were dehydrated in a 30% sucrose solution for 48 h and then embedded in OCT compound (BL557A, Biosharp, China). Sagittal sections (16 μm) of spinal cords were collected using a freezing microtome (NX70, Thermo Fisher, USA). Spinal cord sections were blocked in 5% donkey serum in PBS containing 0.3% Triton X-100 (T8200, Solarbio, China) for 1 h at room temperature and then incubated with primary antibodies in 1% donkey serum containing 0.3% Triton X-100 overnight at 4°C. The primary antibodies used were as follows: goat anti-CD31(1:100, AF3625, R&D Systems), rat anti-PDGFRβ (1:100, 14-1402-82, Invitrogen), goat anti-PDGFRβ (1:100, AF1042, R&D Systems), rabbit anti-NG2 (1:100, AB5320, Sigma-Aldrich), rabbit anti-FSP1 (1:100, 16105-1-AP, Proteintech), rabbit anti-Vimentin (1:300, ab92547, Abcam), rabbit anti-PDGF-BB (1:50, NBP1-58279, Novus), rat anti-GFAP (1:200, 13-0300, Invitrogen), rabbit anti-GFAP (1:100, 16825-1-AP, Proteintech), rat anti-CD68 (1:300, MCA1957, AbD Serotec), goat anti-5-HT (1:5000, #20080, Immunostar), rabbit anti-neurofilament-heavy polypeptide (NF-H, 1:500, ab207176, Abcam), rabbit anti-growth-associated protein 43 (GAP43, 1:100, 16971-1-AP, Proteintech), rabbit anti-NeuN (1:500, ab177487, Abcam), rabbit anti-fibronectin (1:100, 15613-1-AP, Proteintech), rabbit anti-Laminin (1:100, 23498-1-AP, Proteintech), rat anti-Ki67 (1:100, 14-5698-80, Invitrogen), rabbit anti-cleaved caspase-3 (Asp175) (C-Cas3,1:250, 9661, Cell Signaling Technology), and rabbit anti-fibrinogen (1:100, 15841-12-AP, Proteintech). Secondary antibodies, including Alexa Fluor 488 (1:500, A-21206, A-21208, A-11055, Invitrogen) and Alexa Fluor 594 (1:500, A-21209, A-21207, Invitrogen), were used to incubate the sections for the next day. 4′,6-Diamidino-2-phenylindole, dilactate (DAPI, P0126, Beyotime Biotechnology, China) was used for nuclear staining. Immunofluorescence images were acquired with an inflorescent microscope system (Axio Scope A1, Zeiss, Germany) and a confocal microscope (LSM 900, Zeiss, Germany). Images were created by Zen 3.3 software (Blue edition).

### Quantitative analysis

To quantify the microvessel area and fibrotic scar area in the injury site, 3–5 sagittal sections in 4 animals per time point were stained for CD31 and PDGFRβ. The area of CD31^+^ microvessels was measured by ImageJ/Fiji (version 2.3.011/1.53f51) as previously described [22]. The CD31^+^ area was measured using images from ×10 magnification with the Zeiss Axio Scope A1 and is expressed as the proportional area occupied by CD31 staining in tissue sections. For the quantification of fibrotic scar area, immunoreactivities of PDGFRβ, FSP1, and vimentin were measured through threshold processing, and the areas covered by threshold regions were calculated. To quantify the percentage of PDGFRβ^+^ pericyte-associated microvessels and the percentage of NG2^+^PDGFRβ^+^ pericytes, the PDGFRβ^+^ pericytes covering microvessels and NG2^+^PDGFRβ^+^ pericytes were counted, and the data are expressed as a percentage of the total PDGFRβ^+^ pericytes.

To quantify regenerated 5-HT axons growing up at the injured site, 3–5 sagittal sections in 5–7 animals per group were stained for GFAP and 5-HT. The distance from the tip of the 5-HT axon to the edge of the astrocyte scar was measured using ImageJ/Fiji. To assess the density of fibers growing up at the lesion site, threshold regions of NF-H and GAP43 were measured through threshold processing. For the quantification of the number of NeuN^+^ cells, 3–5 sagittal sections in 5–7 animals per group were stained with GFAP and NeuN. The sagittal sections were divided into three zones based on their immediate proximity to the lesion core. These zones were composed of Z1 (0–250 μm), Z2 (250–500 μm), and Z3 (1000–1250 μm) according to a previous description [23]. The number of NeuN^+^ cells was counted using ImageJ/Fiji and expressed as per mm^2^.

To compare differences between the control group and the imatinib group in fibrotic scar formation, inflammatory response, and BSCB leakage, 3–5 sagittal sections per animal were stained for GFAP, PDGFRβ, fibronectin, laminin, FSP1, CD68, and fibrinogen. The area was assessed through threshold processing and measured using ImageJ/Fiji software. The calculated data are expressed as a percentage of the total area. For the quantification of pericyte coverage, 3 sagittal sections in 4–6 animals per group were stained for CD31 and PDGFRβ. Pericyte coverage was measured using images from ×10 magnification with the Zeiss Axio Scope A1 and is presented as the percentage of CD31^+^ microvessels covering the PDGFRβ^+^ pericyte surface out of the total CD31^+^ microvessels. To determine the proliferation and apoptosis of PDGFRβ^+^ cells in the control group and the imatinib group, Ki67-, C-Cas3-, and PDGFRβ-expressing cells at the lesion core were counted in 3–5 sagittal sections in 5–6 animals per group at ×40 magnification and are presented as a percentage. To determine pericyte coverage on microvessels, the number of microvessels covered by PDGFRβ^+^ pericytes was counted and expressed as a percentage.

### Cell culture

A mouse brain microvascular endothelial cell line (bEnd.3, TCM40) was purchased from the National Collection of Authenticated Cell Cultures (Shanghai, China) and cultured in DMEM (12100046, Gibco) supplemented with 10% fetal bovine serum (FBS, 10270106, Gibco) and 1% penicillin-streptomycin (C0222, Beyotime). bEnd.3 cells have been confirmed to have similar morphological and functional characteristics to primary microvascular endothelial cells [24]. Primary mouse brain vascular pericytes (M1200) were obtained from ScienCell^TM^ and cultured in pericyte medium (#1231, ScienCell^TM^) for experiments before passage 5. bEnd.3 cells and pericytes were all identified by short tandem repeat profiling and incubated in a humidified atmosphere (37°C, 5% CO_2_).

### Cell treatment and small interfering RNA (siRNA) transfection

bEnd.3 cells were treated with 1 mg/ml myelin debris for the indicated time points or the indicated concentrations of myelin debris for 72 h. The cells were washed with PBS to remove excess myelin debris and replaced with serum-free medium for 24 h. The supernatants were centrifuged at 1500 rpm for 10 min for ELISA detection. The protein was extracted from the treated bEnd.3 cells to detect the expression level of PDGF-BB. The conditioned medium extracted from the bEnd.3 cells treated with PBS or 1 mg/ml myelin debris for 72 h was named endothelial cell conditioned medium (EC-CM) and myelin debris-induced conditioned medium (Mye-CM), respectively. Primary pericytes were treated with culture medium containing 2% FBS, conditioned medium (EC-CM or Mye-CM) mixed in a 1:1 ratio to pericyte culture medium, or 250 ng/ml PDGF-BB (220-BB-050, R&D Systems) for 72 h. For PDGFRβ blocking experiments, pericytes were pretreated with imatinib (a selective inhibitor of PDGFRβ, 1 mg/ml, HY-590946, MCE) or Su16f (a special inhibitor of PDGFRβ, 10 μm, 3304, R&D Systems) for 12 h before treatment with Mye-CM. siRNA targeting mouse PDGF-BB and negative control siRNA were obtained from GenePharma (Shanghai, China). siRNA targeting PDGF-BB#1 was 5′-UCCGGAGUCGAGUUGGAAATT-3′, PDGF-BB#2 was 5′-GGUGAGAAAGAUUGAGAUUTT-3′, and PDGF-BB#3 was 5′-GCAAGCACCGAAAGUUUAATT-3′. jetPRIME transfection reagent (114–15, Polyplus Transfection) was used to transfect bEnd.3 cells according to the manufacturer’s protocols.

### ELISA detection of PDGF-BB concentration

The PDGF-BB concentration was measured by a mouse PDGF-BB ELISA kit (KE10034, Proteintech, China) according to the manufacturer’s instructions. In brief, the supernatant was extracted from the cell medium and then centrifuged at 1500 rpm for 10 min. Then, 100 μl of supernatant was added to a 96-well microplate and reacted with diluent antibody solution, diluent HRP solution, and TMB substrate. After the reaction was terminated by the stopping solution, the absorbance at 450 nm was measured under an enzyme label instrument (VL0000D0, Thermo Fisher, USA). The absorbance at 630 nm was taken as the calibration value. The standard curve was determined by regression analysis of four-parameter logical curve fitting.

### Western blot

Proteins were isolated from bEnd.3 cells and pericytes and quantified with a BCA kit (BL521A, Biosharp, China). Protein samples (20 μl, 30 μg) were separated by SDS-PAGE and then transferred to PVDF membranes. The membranes were blocked with 5% nonfat milk for 2 h and then probed with primary antibodies overnight at 4°C. The primary antibodies used were as follows: mouse anti-PDGF-BB (1:100, SC-365805, Santa Cruz), rabbit anti-NG2 (1:1000, AB5320, Sigma-Aldrich), rabbit anti-FSP1 (1:1000, 16105-1-AP, Proteintech), rabbit anti-Vimentin (1:6000, 10366-1-AP, Proteintech), mouse anti-Collagen I (1:5000, 67288-1-Ig, Proteintech), rabbit anti-Laminin (1:1000, 23498-1-AP, Proteintech), and mouse anti-β-Tubulin (1:10000, T0023, Affinity). Membranes were incubated with the corresponding HRP-conjugated secondary antibodies (1:10000, A4416, A0545, Sigma-Aldrich). The protein signals were visualized by the Tanon system (5200, Tanon) and then quantified by ImageJ/Fiji.

### Immunocytochemistry

Cell immunocytochemistry was performed in accordance with our previous description [25]. In brief, cells were fixed with 4% PFA, permeabilized with 0.5% Triton X-100 in PBS and blocked with 5% donkey serum in PBS. The primary antibodies and secondary antibodies were used as described above. Immunofluorescence images were obtained in five random fields per sample using an inflorescent microscope system (Axio Scope A1, Zeiss, Germany).

### Imatinib intrathecal injection

Imatinib (HY-590946, MCE) was intrathecally injected at a dose of 10 mg/kg once daily from 4 h until 28 days post-injury (dpi). The animals in the control group were intrathecally injected with the same volume of sterile PBS daily.

### Motor behavioral analysis

All mice received test adaptation training 1 h before behavioral analysis. The tests were performed blindly by two experienced researchers. Basso mouse scale (BMS) score evaluation was performed at 0, 1, 3, 7, 14, 21, and 28 dpi according to an open-field rating scale. The score ranges from 0 to 9. The final score of each mouse was the average of the scores from the two researchers. For footprint analysis, hindlimb locomotor evaluation was performed at 28 dpi. After applying paint to the fore and hind limbs, the mouse walked on a channel 80-cm long and 4-cm wide. Stride length, stride width, and paw rotation were analyzed to evaluate motor function.

### Statistical analysis

All data are presented as the mean ± standard error of the mean (s.e.m) from at least three independent experiments or at least 3 animals per group. One-way analysis of variance (ANOVA) and two-way ANOVA followed by Tukey’s post hoc test or Bonferroni post hoc correction were performed to detect differences among multiple groups at the indicated time points using GraphPad Prism 7.0a software. The two-tailed Student’s *t* test was used to compare two groups. The analysis was determined to be statistically significant at *p*<0.05.

## Results

### Pericytes accumulate in the injury core and transition into fibroblasts to form fibrotic scar after SCI

**Fig. 1:**
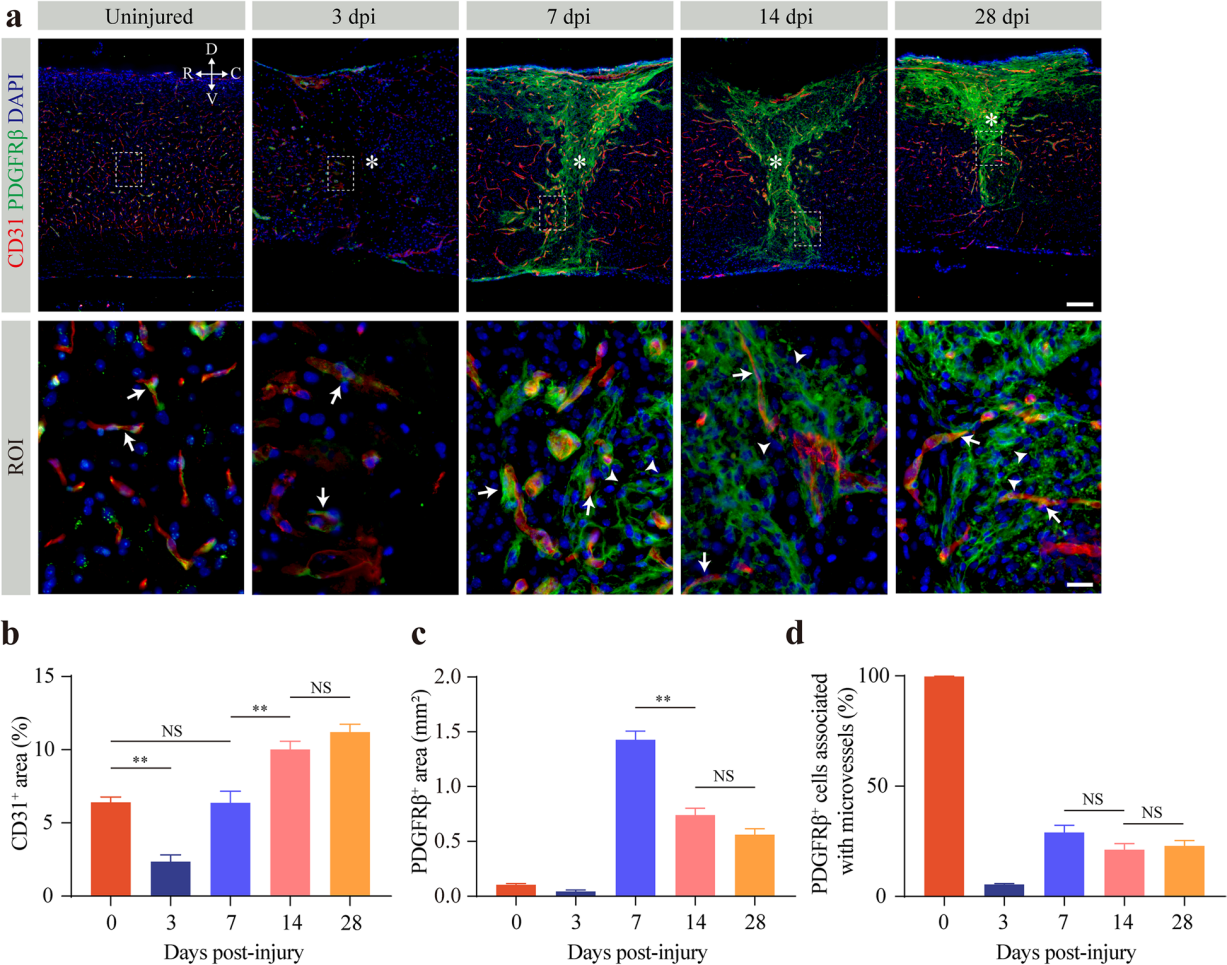
Pericytes detach from the vascular wall and contribute to fibrotic scarring after SCI. **a** Representative immunofluorescence images of microvessels (CD31, red), pericytes (PDGFRβ, green), and DAPI (blue) in the spinal cord of uninjured mice and injured mice at 3, 7, 14, and 28 days post-injury (dpi). High magnification images of the dotted area are shown below as the region of interest (ROI). The asterisks denote the lesion core. PDGFRβ^+^ pericytes are tightly connected (arrows) with or detached from (arrowheads) the vascular wall. Scale bars: 100 μm (upper panel) and 20 μm (lower panel). All images are from sagittal sections. **b–d** Quantification of the percentage of CD31^+^ microvessel area (**b**), PDGFRβ^+^ pericyte area (**c**), and PDGFRβ^+^ cells associated with microvessels (**d**) in the lesion core after SCI. *n* = 4 per time point. Data are shown as mean ± s.e.m. NS, no significance; **p < 0.01 by one-way ANOVA followed by Tukey’s post hoc test in **b**–**d**. D, V, R, and C represent the dorsal, ventral, rostral, and caudal sides of the injury, respectively

To assess the dynamics of fibrotic scarring after SCI, we first determined the spatiotemporal relationship between CD31^+^ microvessels and PDGFRβ^+^ pericytes using immunostaining in uninjured mice and injured mice at 3, 7, 14, and 28 dpi. In the uninjured mice, PDGFRβ^+^ pericytes were tightly associated with microvessels (Fig. 1a). There was a 63.19% decrease in CD31^+^ area and a 59.36% decrease in the PDGFRβ^+^ area in the injured core at 3 dpi relative to the normal spinal cord (Fig. 1b, c). At this time point, the pericytes around the injured core were still tightly connected to the microvessels (Fig. 1a). However, most PDGFRβ^+^ pericytes detached from the blood vessel wall and dispersed in the injured core to form fibrotic scar at 7 dpi (Fig. 1a), which was consistent with previously reported results [3]. The area of newly generated microvessels was similar to that of the uninjured spinal cord, while the area of fibrotic scar peaked at that time (Fig. 1b, c). Over time, the microvessel area of the injured core was 1.57-fold and 1.76-fold higher at 14 dpi and 28 dpi, respectively, than at 7 dpi (Fig. 1b). Accordingly, the area of fibrotic scar decreased by 48.20% and 60.65% at 14 dpi and 28 dpi (Fig. 1c), respectively, compared to 7 dpi, indicating that fibrotic scar was gradually compacted with maturation in the chronic phase of SCI. Despite continued pathological angiogenesis and gradual compaction in fibrotic scar, there was no difference in microvessel area, fibrotic scar area, or the percentage of pericytes associated with microvessels in the lesion core between 14 dpi and 28 dpi (Fig. 1b–d). These findings indicate that the detachment of stromal pericytes from the blood vessel wall is involved in fibrotic scarring accompanied by persistent pathological angiogenesis.

**Fig. 2:**
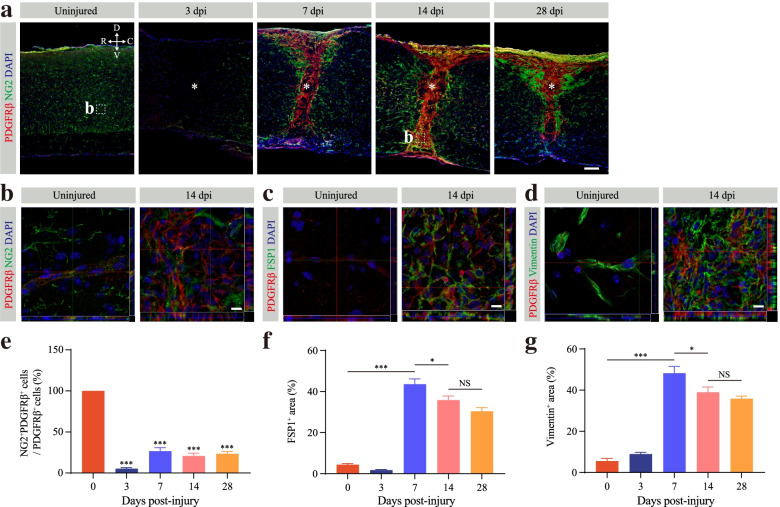
Pericyte transition into fibroblasts after SCI. **a**–**d** Representative immunofluorescence images taken in the spinal cords of uninjured mice and injured mice at 3, 7, 14, and 28 days post-injury (dpi) showing that fibrotic scarring PDGFRβ^+^ fibroblasts (red) lose the expression of the pericyte marker NG2 (green, **a** and **b**) but robustly express the fibroblast markers FSP1 (green, **c**) and vimentin (green, **d**) after SCI. The nuclei are stained with DAPI (blue). The high magnification z-stack images of the dotted area in a are shown below as the region of interest in b. **e** Quantification of the percentage of NG2^+^PDGFRβ^+^ cells out of the total PDGFRβ^+^ cells in the lesion core. **f**, **g** Quantification of the percentage of FSP1^+^ area (**f**) and vimentin^+^ area (**g**). The asterisks indicate the lesion core. Data are shown as mean ± s.e.m. *n* = 4 mice per time point. Scale bars: 100 μm (**a**) and 10 μm (**b–d**). All images are from sagittal sections. NS, no significance; *p < 0.05, ***p < 0.001 by one-way ANOVA followed by Tukey’s post hoc test in **e**, **f**. 3, 7, 14, and 28 dpi versus 0 dpi in **e**

PDGFRβ^+^ pericytes detached from the blood wall can express fibroblast markers such as α-SMA and fibronectin through tracing pericyte fate lineage after SCI [3]. Although the process of pericyte-fibroblast transition has not been confirmed, fibrotic scar-forming cells are all specifically marked by PDGFRβ [3]. Therefore, we used the well-established PDGFRβ marker, combined with its spatial localization away from the blood vessel and located in the lesion core, to characterize and quantitatively analyze fibrotic scar. The phenotypic characteristics of PDGFRβ^+^ pericytes were determined through staining for the pericyte marker NG2 and the fibroblast markers FSP1 and vimentin to further verify the transition process after SCI. Pericytes involved in fibrotic scarring were noted to exhibit a fibroblast phenotype after SCI (Fig. 2a–d). In the uninjured spinal cord, PDGFRβ^+^ pericytes all expressed NG2 proteoglycan and vimentin, whereas PDGFRβ^+^ pericytes did not express FSP1 (Fig. 2a–d). However, few PDGFRβ^+^ cells colocalized with NG2, and NG2 was mainly distributed around PDGFRβ^+^ pericytes at 7, 14, and 28 dpi (Fig. 2a, b). The percentage of NG2^+^ PDGFRβ^+^ cells to the total PDGFRβ^+^ cells was nearly 20% at 7, 14, and 28 dpi, which was significantly lower than that in the uninjured spinal cord (Fig. 2e). Conversely, a large number of PDGFRβ^+^ pericytes abundantly expressed the fibroblast markers FSP1 and vimentin at 14 dpi, which indicated that pericytes acquired a fibroblast phenotype (Fig. 2c, d). The fibroblast phenotype persisted until the fibrotic scar matured at 28 dpi (Supplementary Fig. 1a, b). Notably, vimentin was also expressed in astrocytes after SCI [26]. Nonetheless, our further analysis showed that the areas of FSP1 and vimentin peaked at 7 dpi (Fig. 2f, g). As the fibrotic scar matured, the fibrotic area decreased significantly at 14 dpi, and there was no significant difference at 14 and 28 dpi after SCI (Fig. 2f, g). Therefore, PDGFRβ^+^ pericytes gain a stromal fibroblast phenotype in the lesion core after SCI. Together, these results suggest that pericytes detach from the blood vessel wall and transition into stromal fibroblasts after SCI.

### Microvascular endothelial cells induce pericyte-fibroblast transition through the PDGF-BB/PDGFRβ signaling pathway

**Fig. 3:**
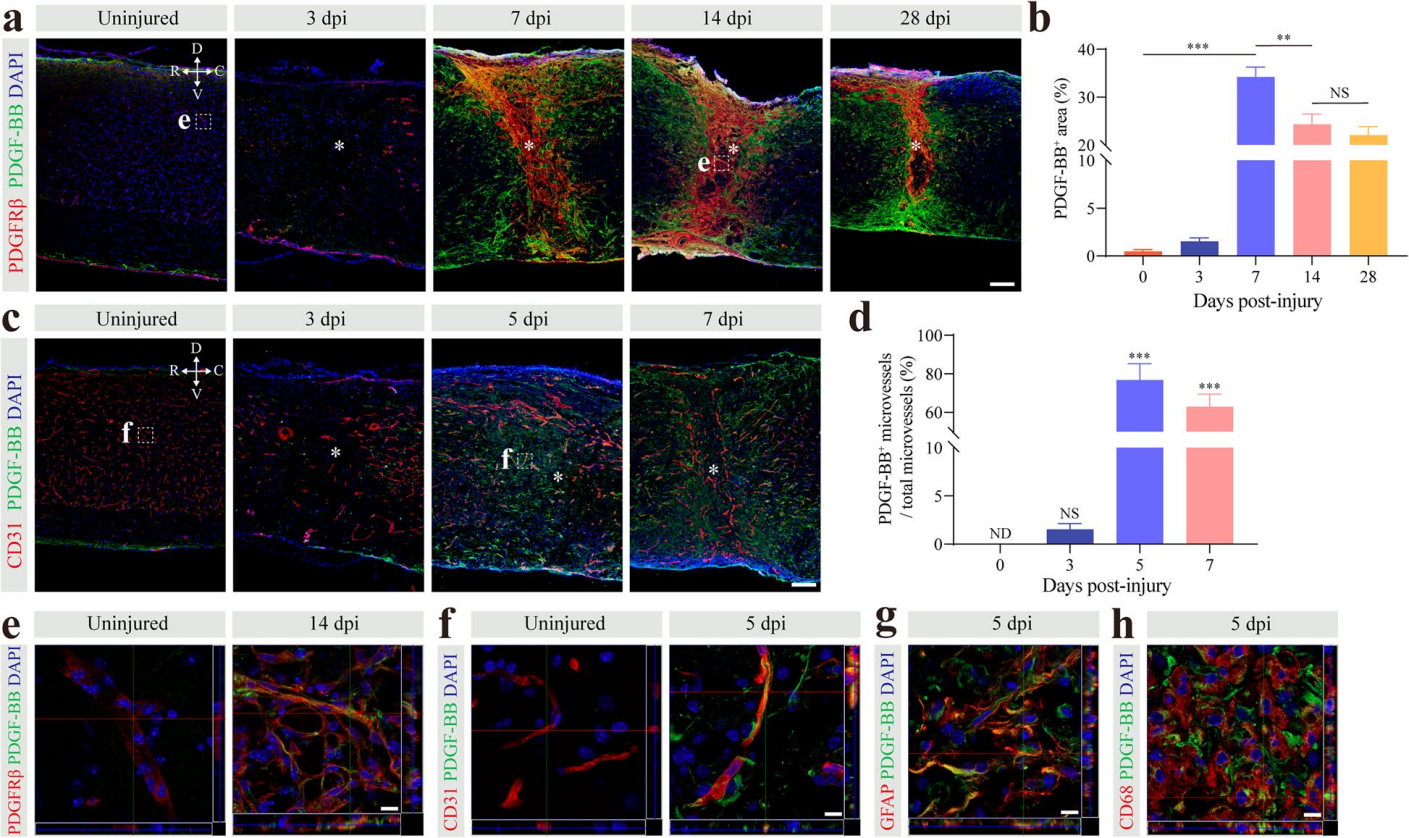
PDGF-BB is mainly distributed around the injury core and is of various cellular origins. **a** Representative immunofluorescence images taken in the spinal cord of uninjured mice and injured mice at 3, 7, 14, and 28 days post-injury (dpi) showing that PDGF-BB (green) is predominantly distributed around the lesion core and closely correlated to the spatiotemporal distribution of PDGFRβ (red). **b** Quantification of the percentage of PDGF-BB^+^ area. **c** Representative immunofluorescence images of microvascular endothelial cells (CD31, red) and PDGF-BB (green) in the spinal cord of uninjured mice and injured mice at 3, 5, and 7 dpi. **d** Quantification of the percentage of PDGF-BB^+^ microvessels out of the total microvessels. **e**, **f** High magnification z-stack images of the dotted area in **a** and **c** are shown below as the region of interest in **e** and **f**, respectively. The lesion core is marked with asterisks. **g**, **h** Representative immunofluorescence images of GFAP^+^ astrocytes (red, **g**) and CD68^+^ microglia/macrophages (red, **h**) with PDGF-BB (green) at 5 dpi. The nuclei are stained with DAPI (blue). Data are shown as mean ± s.e.m. *n* = 4 mice per time point. Scale bars: 100 μm (**a** and **c**) and 10 μm (**e**–**h**). All images are from sagittal sections. ND, not determined; NS, no significance; **p < 0.01, ***p < 0.001 by one-way ANOVA followed by Tukey’s post hoc test in **b** and **d**. 3, 5, and 7 dpi versus 0 dpi in **d**

The PDGF-BB/PDGFRβ signaling pathway plays an essential role in the pericyte-fibroblast transition under pathological fibrosis [11]. To clarify the mechanism of pericyte-fibroblast transition after SCI, we detected PDGF-BB and PDGFRβ expression in the uninjured mice and injured mice at 3, 7, 14, and 28 dpi. In the uninjured spinal cords, PDGF-BB and PDGFRβ were not colocalized (Fig. 3a, e). By 7, 14, and 28 dpi, PDGF-BB was mainly distributed in the penumbra area of the injury site and at the periphery of PDGFRβ^+^ cells (Fig. 3a). We found that PDGF-BB and PDGFRβ had similar time-dependent patterns, and their areas both peaked at 7 dpi (Fig. 3b). The distribution of PDGF-BB after SCI was consistent with the position of the pericyte-fibroblast transition, which indicated that the PDGF-BB/PDGFRβ signaling pathway may mediate the pericyte-fibroblast transition. In the normal central nervous system, PDGF-BB is mainly derived from microvascular endothelial cells [16, 17]. Pericyte-fibroblast transition began at 3 dpi, and newly generated microvessels appeared in the injury core as early as day 3 after injury. At this time, astrocytes, macrophages, and microglia are not yet involved in wound healing after SCI. Therefore, we further examined the expression of PDGF-BB in microvascular endothelial cells. We found that PDGF-BB was expressed in microvascular endothelial cells at 5 dpi and 7 dpi using immunostaining (Fig. 3c, f). The PDGF-BB expression level after SCI was significantly higher than that in the uninjured spinal cord (Fig. 3d). In addition, we observed that PDGF-BB was also expressed in GFAP^+^ astrocytes and CD68^+^ macrophages/microglia at 5 dpi (Fig. 3g, h). Taken together, these findings indicate that PDGF-BB is mainly distributed around the injury core and that microvascular endothelial cells are one of the cellular sources.

**Fig. 4:**
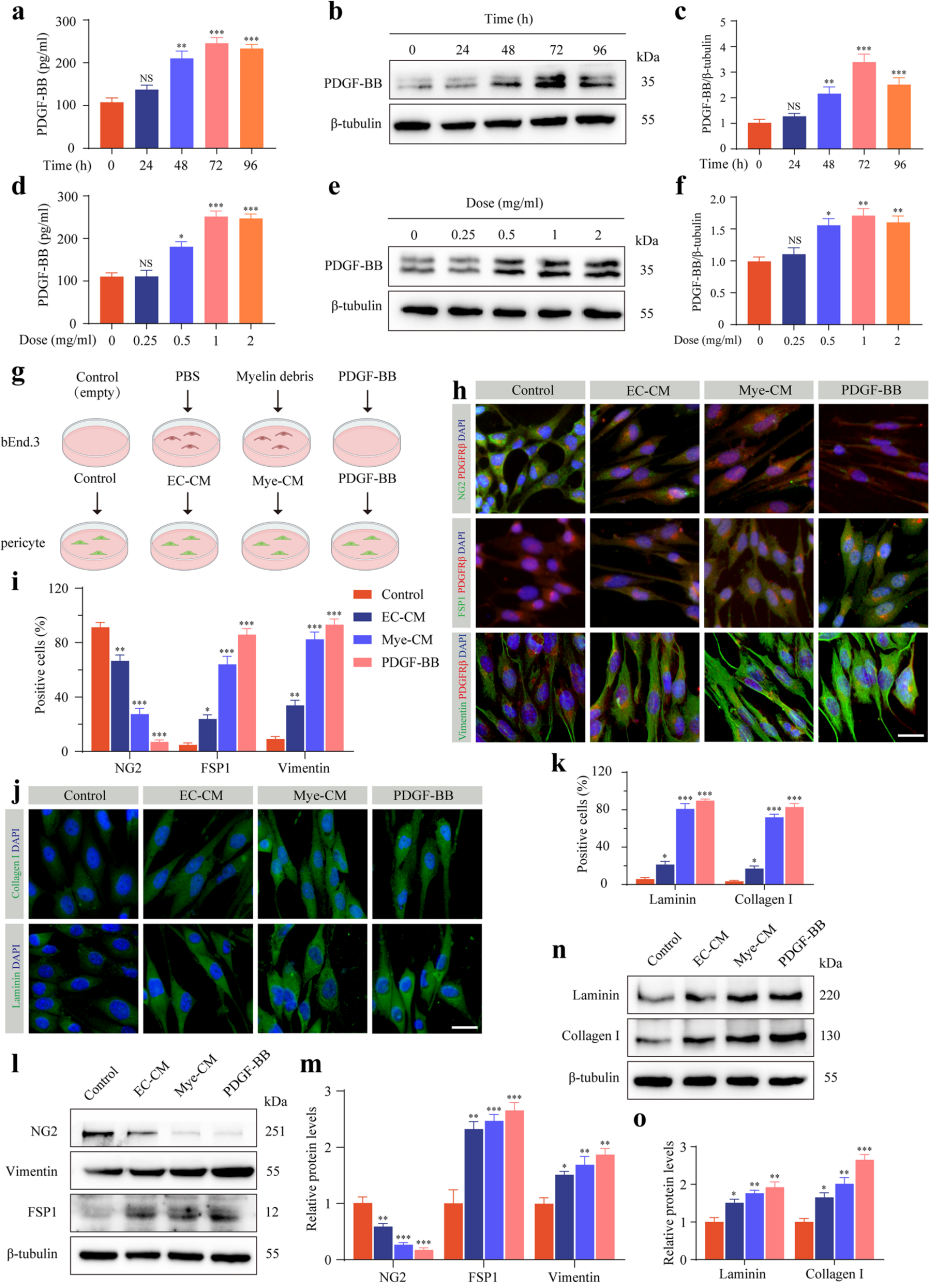
Endothelial PDGF-BB induces pericyte-fibroblast transition and extracellular matrix deposition in vitro. **a** The PDGF-BB expression levels in bEnd.3 cells treated with 1 mg/ml myelin debris at the indicated time points were measured by ELISA. **b**, **c** Western blot analysis (**b**) and quantification (**c**) of PDGF-BB in bEnd.3 cells treated as described above in **a**. **d** The PDGF-BB expression levels in bEnd.3 cells treated with the indicated concentrations of myelin debris for 72 h were measured by ELISA. **e**, **f** Western blot analysis (**e**) and quantification (**f**) of PDGF-BB in bEnd.3 cells treated as described above in **d**. **g** Experimental schematic diagram of pericyte phenotypic transition induced by culture medium (empty, control), EC-CM treated with PBS, Mye-CM treated with myelin debris, and PDGF-BB. **h** Immunostaining of PDGFRβ (red), pericyte marker NG2 (green, upper panel), and fibroblast markers FSP1 (green, middle panel) and vimentin (green, lower panel) in primary pericytes treated as in **g**. **i** Quantification of the percentage of NG2^+^, FSP1^+^, and vimentin^+^ pericytes. **j**, **k** Immunostaining and quantification of extracellular matrix collagen I (green, upper panel) and laminin (green, lower panel) in primary pericytes treated as described in **g**. The nuclei are stained blue with DAPI. **l**, **m** Western blot analysis (**l**) and quantification (**m**) of NG2, FSP1, and vimentin in primary pericytes treated as described above. **n**, **o** Western blot analysis (**n**) and quantification (**o**) of extracellular matrix collagen I and laminin in primary pericytes treated as described above. Scale bar: 25 μm (**h** and **j**). Data are expressed as mean ± s.e.m. *n* = 3 independent cultures. *p < 0.05, **p < 0.01, and ***p < 0.001 versus 0 h, 0 mg/ml or control by one-way ANOVA followed by Tukey’s post hoc test in **a**, **c**, **d**, **f**, **i**, **k**, **m**, and **o**

Microvascular endothelial cells act as amateur phagocytes to engulf myelin debris to promote robust angiogenesis and fibrosis [12]. To investigate whether microvascular endothelial PDGF-BB mediates pericyte-fibroblast transition after SCI, we cultured bEnd.3 cells with the indicated concentrations of myelin debris or for the indicated time points and then extracted conditioned medium to incubate primary pericytes in vitro. Myelin debris treatment in bEnd.3 cells resulted in a higher level of PDGF-BB expression for 72 h at a concentration of 1 mg/ml by ELISA detection and Western blot assay (Fig. 4a–f). To test the interaction between microvascular endothelial cells and pericytes, we examined the effect of bEnd.3 cells treated with PBS or myelin debris on primary pericytes (Fig. 4g). After the primary pericytes were cultured with Mye-CM, the pericytes lost the marker NG2 and expressed a higher level of the fibroblast markers FSP1 and vimentin (Fig. 4h, i), indicating that pericytes underwent pericyte-fibroblast transition. In addition, Mye-CM-cultured pericytes expressed higher levels of extracellular matrix collagen I and laminin (Fig. 4j, k). The significant reduction in the pericyte marker NG2, increase in the fibroblast markers FSP1 and vimentin, and promotion of extracellular matrix collagen I and laminin expression were further confirmed by Western blot assay after Mye-CM stimulation (Fig. 4l–o).

**Fig. 5:**
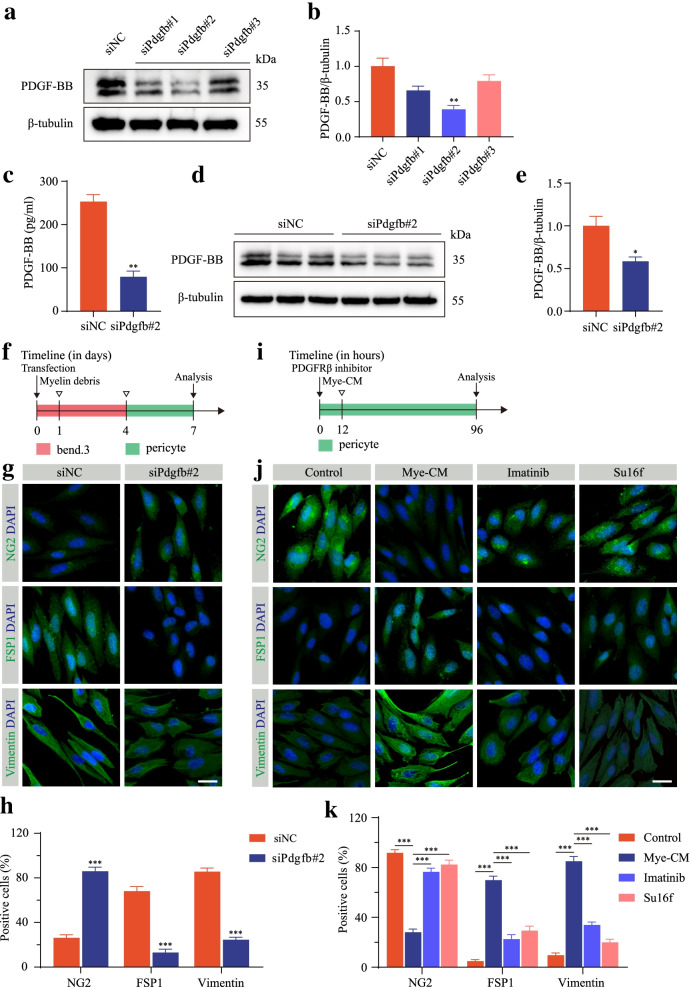
Microvascular endothelial cells induce pericyte-fibroblast transition via the PDGF-BB/PDGFRβ signaling pathway in vitro. **a**, **b** Western blot analysis (**a**) and quantification (**b**) of PDGF-BB in bEnd.3 cells transfected with siNC or siRNAs targeting Pdgfb. **c** The expression levels of PDGF-BB in bEnd.3 cells transfected with siNC or siPdgfb#2 followed by myelin debris treatment were detected by ELISA. **d**, **e** Western blot analysis (**d**) and quantification (**e**) of PDGF-BB in bEnd.3 cells treated as described above in **c**. **f** Experimental schematic diagram of pericyte phenotypic transition transfected with siNC or siPdgfb#2 followed by myelin debris treatment. **g** Immunostaining of NG2 (green, upper panel), FSP1 (green, middle panel), and vimentin (green, lower panel) in primary pericytes treated as described above in **f**. **h** Quantification of the percentage of NG2^+^, FSP1^+^, and vimentin^+^ pericytes in **g**. **i** Experimental schematic diagram of pericyte phenotypic transition blocked with the PDGFRβ inhibitor imatinib (a selective PDGFRβ inhibitor) or Su16f (a specific PDGFRβ inhibitor) followed by Mye-CM. **j** Immunostaining of NG2 (green, upper panel), FSP1 (green, middle panel), and vimentin (green, lower panel) in primary pericytes treated as described in **i**. **k** Quantification of the percentage of NG2^+^, FSP1^+^, and vimentin^+^ pericytes in **j**. Scale bars: 25 μm (**g** and **j**). Data are expressed as mean ± s.e.m. *n* = 3 independent cultures. **p < 0.01 and ***p < 0.001 by one-way ANOVA followed by Tukey’s post hoc test in **b** versus siNC, and **k**. *p < 0.05, **p < 0.01, and ***p < 0.001 versus siNC by unpaired two-tailed Student’s t test in **c**, **e**, and **h**

We next reasoned whether PDGF-BB was required for microvascular endothelial cell-induced pericyte-fibroblast transition. bEnd.3 cells were transfected with siRNAs targeting PDGF-BB (siPdgfb#1, siPdgfb#2, and siPdgfb#3) and the negative control (siNC). The knockdown effectiveness was assessed by Western blot assay (Fig. 5a, b). The expression level of PDGF-BB in the culture medium detected by ELISA was significantly reduced after PDGF-BB knockdown followed by myelin debris treatment (Fig. 5c). The results of Western blot assay further confirmed that PDGF-BB knockdown significantly reduced the PDGF-BB expression level after myelin debris treatment (Fig. 5d, e). To clarify the influence of endothelial PDGF-BB on pericyte-fibroblast transition, bEnd.3 cells were transfected with siPdgfb#2 followed by myelin debris treatment (Fig. 5f). Conditioned medium was used to culture primary pericytes. PDGF-BB knockdown increased the percentage of the pericyte marker NG2 and decreased the percentage of the fibroblast markers FSP1 and vimentin (Fig. 5g, h), indicating that PDGF-BB is required for microvascular endothelial cell-induced pericyte-fibroblast transition in vitro. We next used imatinib and Su16f to block PDGF-BB/PDGFRβ signaling in primary pericytes and then cultured primary pericytes with Mye-CM (Fig. 5i). Both imatinib and Su16f effectively reversed microvascular endothelial cell-induced pericyte-fibroblast transition (Fig. 5j, k). Taken together, these results show that microvascular endothelial cells induce pericyte-fibroblast transition through the PDGF-BB/PDGFRβ signaling pathway.

### Pharmacological inhibition of PDGF-BB/PDGFRβ signaling promotes functional recovery and axonal regeneration

**Fig. 6:**
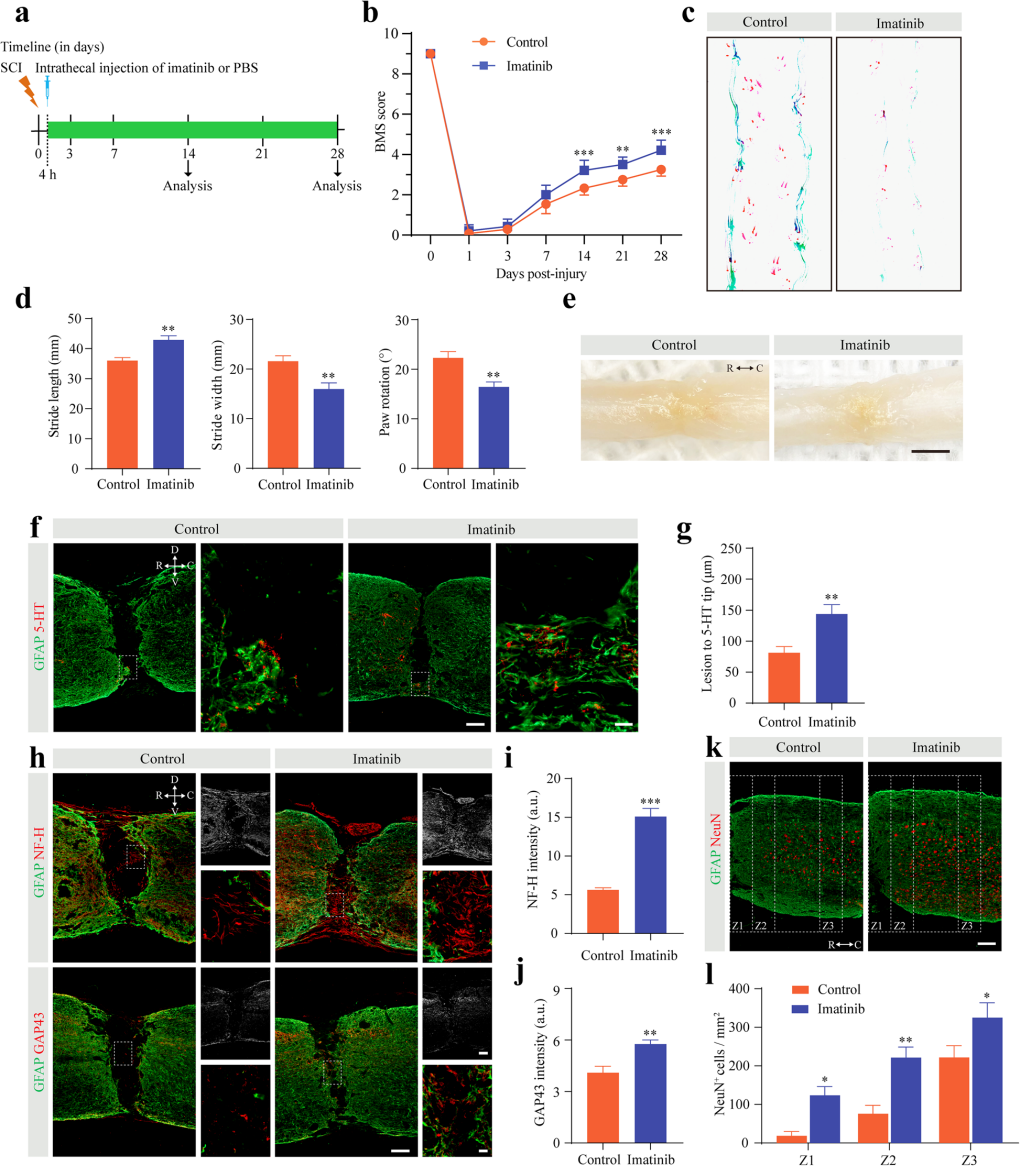
Pharmacologically inhibiting the PDGF-BB/PDGFRβ signaling pathway facilitates functional recovery and axonal regeneration after SCI. **a** Scheme of the experimental setup. Imatinib or PBS was injected intrathecally every day from 4 h until 28 days post-injury (dpi). **b** Behavioral testing using the BMS score was performed at the indicated time points. **c**, **d** Footprint assays (**c**) and quantitative analysis (**d**) in mice treated with PBS or imatinib at 28 dpi. **e** Low-power photographs of intact spinal cord tissue in mice treated as above at 28 dpi. **f** Representative immunofluorescence images of GFAP (green) and 5-HT axons (red) in mice treated as described above at 28 dpi. Right panel show high magnification images in the dotted box on the left panel. **g** Quantification of the lesion distance to the 5-HT axon tip. **h**, **k** Representative immunofluorescence images of GFAP (green), NF-H fibers (red), GAP43 fibers (red), and NeuN^+^ neurons (red) in Z1-Z3 zones adjacent to the lesion core in mice treated as above at 28 dpi. Right panel show brightfield image (upper) and high magnification image (lower) in the dotted box in the left panel of **h**. Scale bars: 200 μm (**e**), 100 μm (left panel in **f** and **h**, right upper panel in **h** and **k**), and 20 μm (right panel in **f** and right lower panel in **h**). **i**, **j**, **l** Quantification of NF-H intensity (**i**), GAP43 intensity (**j**), and NeuN^+^ neurons numbers (**l**). Data are expressed as mean ± s.e.m. *n* = 5–7 per time point or per group. All images are from sagittal sections. *p < 0.05, **p < 0.01, and ***p < 0.001 versus control by two-way ANOVA followed by Bonferroni’s post hoc test in **b** and **l**. **p < 0.01 and ***p < 0.001 versus control by unpaired two-tailed Student’s *t* test in **d**, **g**, **i**, and **j**

To investigate the role of PDGF-BB/PDGFRβ signaling in functional recovery and axonal regeneration after SCI, mice were injected intrathecally daily with imatinib from 4 h after SCI until 28 dpi (Fig. 6a). The mice that received imatinib displayed significantly better recovery in locomotor function than the control mice from 14 to 28 dpi using the BMS score for behavioral analysis (Fig. 6b). In addition, footprint analysis further confirmed that better locomotor function recovery was established at 28 dpi in the imatinib group than in the control group (Fig. 6c, d). There was no obvious spinal cord tissue defect in mice treated with intrathecal injection of imatinib compared to control mice at 28 dpi after SCI (Fig. 6e). These data show that blocking PDGF-BB/PDGFRβ signaling contributes to functional recovery without tissue defects after SCI.

Spinal cord tissue injury disrupts descending motor pathways, and functional recovery implies increased axonal regeneration and fibers. To investigate the influence of inhibiting PDGF-BB/PDGFR signaling on axonal regeneration, we next examined the descending serotonergic 5-HT axon (Fig. 6f), NF-H fibers (Fig. 6h), and GAP43 fibers (Fig. 6h) regeneration using immunostaining at 28 dpi after SCI. There were significantly more 5-HT axon fibers crossing the lesion site and NF-H fibers and GAP43 fibers present in the injury core in the imatinib group than in the control group (Fig. 6g, i, and j). In addition, we used the NeuN antibody, a neuronal marker, to examine neuronal survival in specific zones (Z1-Z3) adjacent to the lesion core [23]. The number of viable NeuN^+^ neurons in the Z1-Z3 region of the spinal cord was significantly increased in imatinib-treated mice compared to control mice (Fig. 6k, l). Altogether, these results indicate that pharmacological inhibition of PDGF-BB/PDGFRβ signaling promotes functional recovery and axonal regeneration after SCI.

### Pharmacological inhibition of PDGF-BB/PDGFRβ signaling reduces fibrotic scarring

**Fig. 7:**
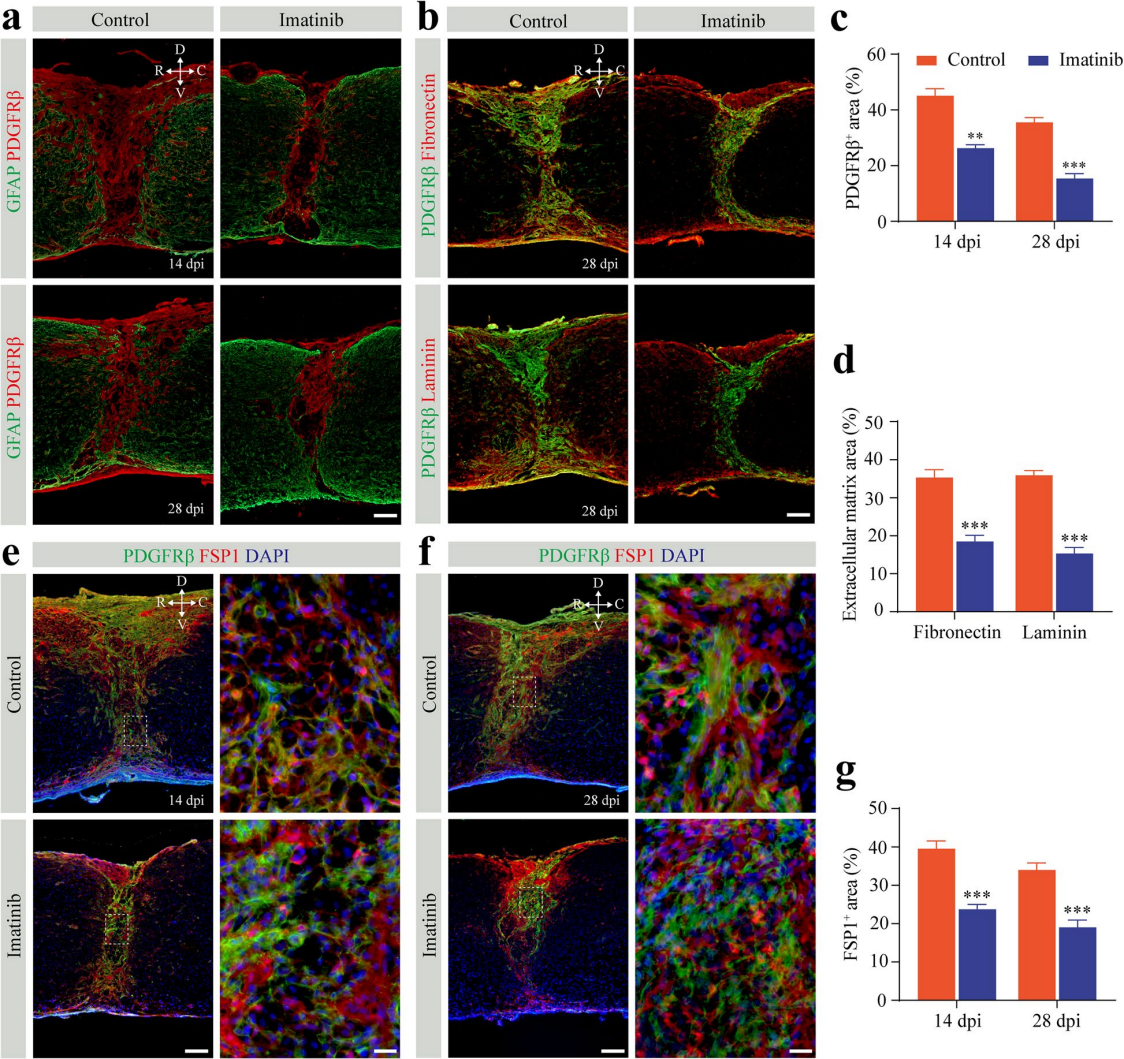
Pharmacologically inhibiting the PDGF-BB/PDGFRβ signaling pathway reduces fibrotic scarring and fibroblasts after SCI. **a** Representative immunofluorescence images of GFAP (green) and PDGFRβ (red) in mice treated with imatinib or PBS (control) at 14 and 28 days post-injury (dpi). **b** Representative immunofluorescence images of PDGFRβ (green) and extracellular matrix fibronectin (red, upper panel) and laminin (red, lower panel) at 28 dpi. **c**, **d** Quantification of fibrotic scar area (**c**) and extracellular matrix area (**d**) in **a** and **b**. **e**, **f** Representative immunofluorescence images of PDGFRβ (green) and FSP1 (red) in mice treated as described above at 14 dpi (**e**) and 28 dpi (**f**). The nuclei are stained with DAPI (blue). High magnification images of the dotted area in the left panel are shown in the right panel. All images are from sagittal sections. **g** Quantification of the fibroblast area occupied by FSP1 in **e** and **f**. Scale bars: 100 μm (**a**, **b** and left panel in **e** and **f**) and 20 μm (right panel in **e** and **f**). Data are expressed as mean ± s.e.m. *n* = 4–6 per group. **p < 0.01 and ***p < 0.001 versus control by unpaired two-tailed Student’s *t* test in **c**, **d**, and **g**

To validate the role of PDGF-BB/PDGFRβ signaling in fibrotic scarring, we examined the pathohistological changes in the lesion site at the time of astrocytic-fibrotic scar boundary establishment 14 dpi and a chronic phase point at 28 dpi with imatinib treatment as described above. Immunohistochemical analysis confirmed that the PDGFRβ^+^ area of fibrotic scar at the lesion core was significantly reduced by 41.81% and 56.74% at 14 dpi and 28 dpi, respectively (Fig. 7a, c). Furthermore, the areas of extracellular matrix fibronectin and laminin were reduced by 47.68% and 57.34% at 28 dpi, respectively, after treatment with imatinib (Fig. 7b, d). Altogether, these data indicate that blocking PDGF-BB/PDGFRβ signaling with imatinib can reduce fibrotic scarring. Importantly, the dense population of FSP1^+^ fibroblasts in the lesion core was markedly reduced both at 14 dpi and 28 dpi after treatment with imatinib compared to that of the control group (Fig. 7e–g), indicating that blocking the PDGFRβ receptor inhibits pericyte-fibroblast transition after SCI. The dramatic change in fibrotic scar area and fibroblast population explains the reason for the functional recovery and axonal regeneration after imatinib treatment. Taken together, these results show that PDGF-BB-PDGFR signaling contributes to fibrotic scar formation.

### Blocking the PDGFRβ receptor inhibits proliferation and promotes apoptosis of PDGFRβ^+^ cells after fibrotic scar formation

Pericytes are activated, transition, and proliferate to form a large number of fibroblasts in the lesion site after SCI [3]. In both the acute and chronic phases after SCI, all fibroblasts that form fibrotic scar express PDGFRβ marker [2]. In the lesion core of SCI, fibroblasts connect to each other to form a typical honeycomb rather than attaching to the microvessels. To further gain more insights into the consequences of PDGFRβ blockade on PDGFRβ^+^ cells after fibrotic scar formation, we detected the difference in PDGFRβ^+^ cells proliferation using a Ki67 antibody and apoptosis using a C-Cas3 antibody at 14 dpi in the mice treated with imatinib or PBS (Supplementary Fig. 2a, b). Notably, we found lower proliferation rates and higher apoptosis rates of PDGFRβ^+^ cells in the mice treated with imatinib than in the control mice (Supplementary Fig. 2c, d). These results indicate that blocking PDGFRβ impairs PDGFRβ^+^ cells survival after fibrotic scarring.

### Pharmacological inhibition of PDGF-BB/PDGFRβ signaling reduces microvessel leakage

**Fig. 8:**
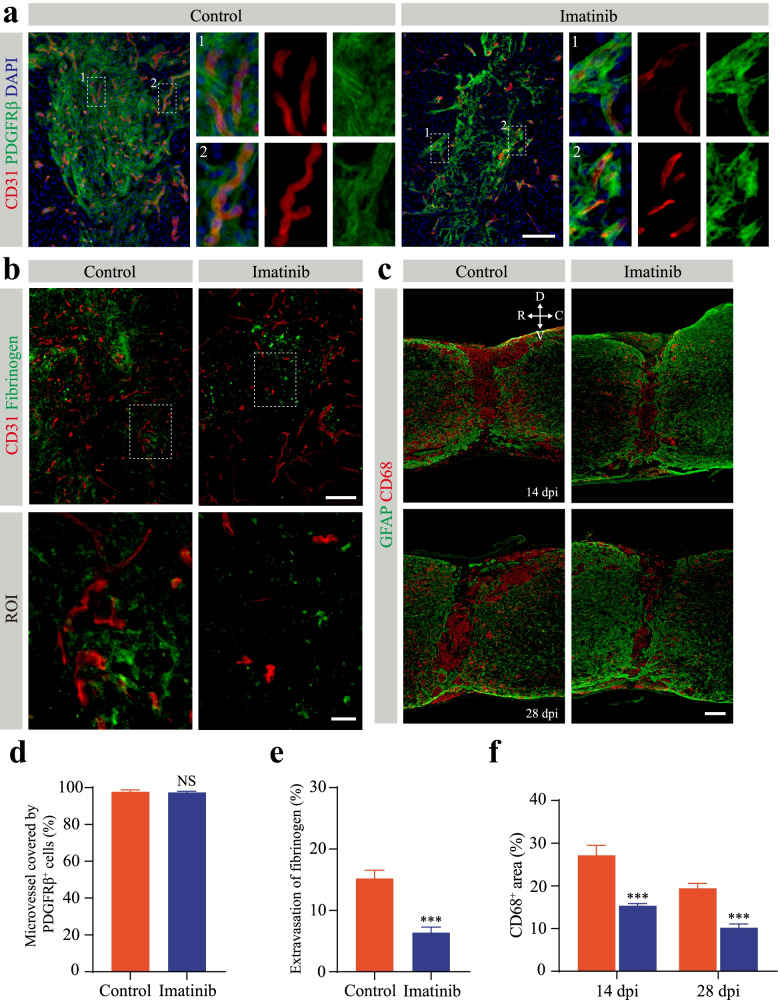
Imatinib has no effect on pericytes covering microvessels but reduces inflammation and microvessel leakage after SCI. **a** Representative immunofluorescence images of CD31 (red), PDGFRβ (green), and DAPI (blue) in mice treated with intrathecal injection of imatinib or PBS (control) at 14 days post-injury (dpi). Enlarged images of the dotted line in the left panel are shown in the right panel. **b** Representative immunofluorescence images of CD31 (red) and fibrinogen (green) in mice treated as above. The lower panel shows high magnification image in the dotted box in the upper panel. **c** Representative immunofluorescence images of GFAP (green) and CD68 (red) in mice treated as described above at 14 dpi (upper panel) and 28 dpi (lower panel). Scale bars: 100 μm (**a**, upper panel in **b** and **c**) and 20 μm (lower panel in **b**). All images are from sagittal sections. **d**–**f** Quantification of the percentage of microvessel covered by PDGFRβ^+^ cells (**d**), fibrinogen leakage (**e**), and the CD68^+^ inflammatory area (**f**) in **a**–**c**. Data are expressed as mean ± s.e.m. *n* = 4–6 per group. NS, no significance; ***p < 0.001 versus control by unpaired two-tailed Student’s *t* test in **d**, **e**, and **f**

Microvessel leakage caused by the breakdown of the BSCB is one of the obstacles for functional recovery after SCI [27]. To evaluate the effect of blocking PDGFRβ on microvessel leakage, we first assessed the pericyte coverage of newly formed microvessels in the lesion core using immunofluorescence staining after imatinib treatment as described above. Pericyte coverage refers to the percentage of microvessels covered by PDGFRβ^+^ pericytes in the lesion core. A large population of PDGFRβ^+^ pericytes were still covered with the blood vessel wall in the lesion core at 14 dpi after treatment with imatinib (Fig. 8a). The pericyte coverage in the SCI lesion core of the mice in the control group and the imatinib treatment group was 97.71±1.08% and 97.38±0.685%, respectively (Fig. 8d). There was no significant difference in the pericyte coverage of newly formed microvessels in the lesion core between the imatinib group and the control group (Fig. 8d). The BSCB is impaired after SCI, and fibrinogen, as the main coagulation protein in plasma and inflammation inducer, leaks into the spinal cord parenchyma to cause an inflammatory response and microvascular remodeling [28, 29]. Immunofluorescence staining of CD31 and fibrinogen was performed after perfusion in the normal mouse spinal cord. No fibrinogen leakage was observed in the spinal cord parenchyma (Supplementary Fig. 3). We next examined the distribution of microvessels and fibrinogen in the lesion site at 14 dpi to eliminate the effect of fibrinogen leakage in the acute phase. By 14 dpi, fibrinogen extravascular leakage was markedly lower after imatinib treatment than that in the control mice (Fig. 8b, e). A large population of macrophages/microglia infiltrate the injured site, and the release of inflammatory factors can lead to increased microvessel leakage [21]. We reasoned that the inflammatory response mediated by leaking fibrinogen led to increased microvessel leakage. The area of CD68^+^ macrophages/microglia at the lesion site was significantly smaller in the imatinib group than in the control group at 14 dpi and 28 dpi (Fig. 8c, f). Quantitation of CD68^+^ macrophage/microglial staining showed that the positive area decreased by 43.54% and 47.58% at 14 dpi and 28 dpi after imatinib treatment compared to that of the control mice, respectively (Fig. 8f). Thus, our data indicate that the reduction in microvessel leakage may be associated with inflammation attenuation after SCI.

## Discussion

**Fig. 9:**
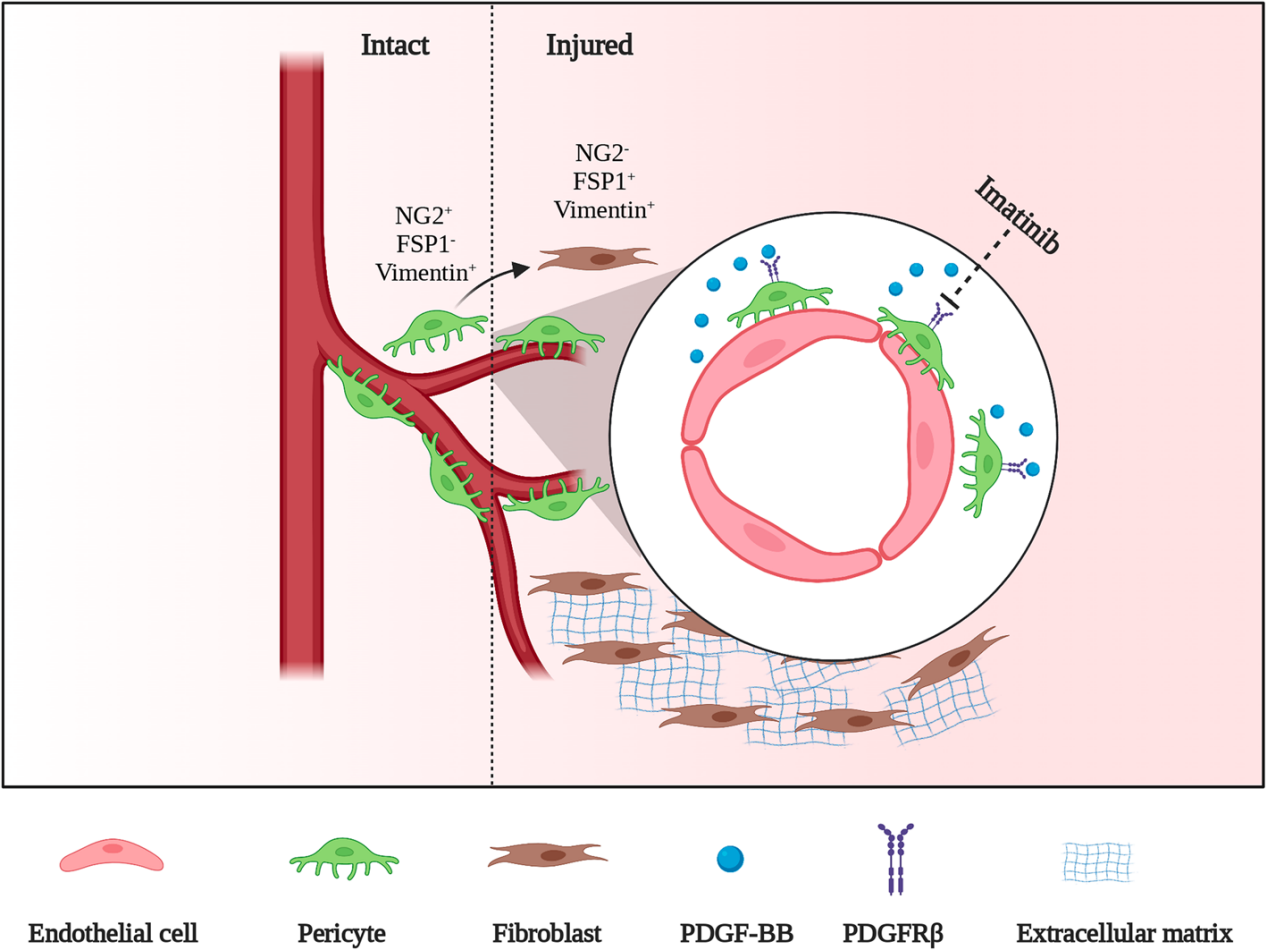
Microvascular endothelial cells secrete PDGF-BB to induce PDGFRβ^+^ pericyte detachment from the blood vessel wall and then transition into fibroblasts, which contributes to the formation of fibrotic scar. The pharmacologic PDGFRβ inhibitor imatinib restrains pericyte-fibroblast transition and fibrotic scar formation

Pericyte-derived stromal fibroblasts form fibrotic scar after SCI [3, 13]. Herein, we reveal that the PDGF-BB/PDGFβ signaling pathway is involved in microvascular endothelial cell-induced pericyte-fibroblast transition (Fig. 9). Pharmacological blockade of the PDGF-BB/PDGFRβ signaling pathway with imatinib is beneficial for promoting functional recovery and axonal regeneration and attenuating fibrotic scarring after SCI. After fibrotic scar formation, imatinib can suppress the survival of PDGFRβ^+^ cells by inhibiting proliferation and promoting apoptosis. In addition, pharmacological blockade of the PDGF-BB/PDGFRβ signaling pathway with imatinib reduces microvessel leakage and inhibits the inflammatory response.

The cellular source of fibroblasts has always been the focus of research in fibrotic scar after SCI [2, 3]. The original source of fibroblasts was thought to be the meningeal cells surrounding the brain and spinal cord. A previous study showed that Glast^+^ type A pericytes are the source of fibroblasts after SCI [3]. The study is based on the recombination of Glast-Cre^ER^ mice to divide pericytes into two distinct types. Type A pericytes detach from the blood vessel wall and form fibrotic scar, whose markers are PDGFRβ and CD13. Type B pericytes connect tightly with the blood vessel wall, and the markers are desmin and/or a-SMA. A subsequent study used Col1α1-GFP mice to track the process of fibrotic scar formation, indicating that Col1α1^+^ cells around large blood vessels are the source of fibroblasts that form fibrotic scar [2]. Col1α1 can be expressed in both pericytes and fibroblasts under normal physiological conditions [11, 17]. Tracking the fate of Col1α2-expressing cells in the experimental allergic encephalomyelitis model revealed that Col1α2^+^ perivascular fibroblasts form fibrotic scar [30]. Neither the Col1α1 reporter line nor the Col1α2 reporter line can avoid marking pericytes while tracking fibroblasts because pericytes and fibroblasts share similar origins during development. Similarly, using the Glast-YFP reporter line to track the fate of pericytes is also unavoidable to label fibroblasts, as a recent single-cell sequencing study showed that Glast could be expressed in a fibroblast cluster [17]. In addition, microvascular endothelial cells engulfing myelin debris undergo endothelial-mesenchymal transformation into fibroblast-like cells, suggesting that microvascular endothelial cells may be another source of fibroblasts [12]. Therefore, the cell sources of fibroblasts after SCI include pericytes, fibroblasts, and microvascular endothelial cells. In this study, we showed that PDGFRβ^+^ perivascular pericyte transition into stromal fibroblasts. PDGFRβ^+^ pericytes no longer expressed NG2 in the injured core after transition into stromal fibroblasts in line with a previous report [13]. We found that a small part of NG2 proteoglycan was expressed in the injured core. The NG2 proteoglycan was expressed in pericytes associated with microvessels and macrophages after phagocytosing myelin debris in the lesion core [31]. The newly formed microvessels were still covered with PDGFRβ^+^ perivascular pericytes in the injured core. Although this portion of pericytes is close to the blood vessel wall, it has obvious heterogeneity with the pericytes in the normal spinal cord. Future investigation is required to assess whether this heterogeneity is associated with the inflammatory response in the injured core. In addition, the fibroblast marker FSP1 could not completely colocalize with PDGFRβ after SCI, which may indicate that fibroblasts may also be derived from local spinal cord fibroblasts.

PDGF-BB is an important regulator in the process of angiogenesis. In this study, we confirmed that the formation of robust fibrotic scar was accompanied by extensive angiogenesis after SCI. In the normal brain, endothelial cells are the primary source of PDG-BB, as confirmed by a single-cell sequencing study [17]. Pericytes with characteristics of mesenchymal stem cells contact microvascular endothelial cells without transitioning into other cell types. We found that microvascular endothelial cells were one of the sources of PDGF-BB after SCI in the acute phase. Microvascular endothelial cells, as amateur phagocytes, can engulf myelin debris and promote fibrosis after SCI [12]. We treated microvascular endothelial cells with myelin debris in vitro and found that the concentration of PDGF-BB was significantly increased. It was obvious that microvascular endothelial-derived PDGF-BB was required for pericyte-fibroblast transition. In fact, the effects of PDGF-BB on pericytes are dichotomous depending on the concentration. A low concentration of PDGF-BB (25 ng/ml) promotes pericyte migration [32], while a high concentration of PDGF-BB (250 ng/ml) promotes pericyte transition into other cells [33]. Hence, microvascular endothelial cells maintain the mesenchymal stem cell properties of pericytes under normal physiological conditions. Using imatinib to block PDGFRβ receptors, we found that microvascular endothelial cells induced pericyte-fibroblast transition through the PDGF-BB/PDGFRβ signaling pathway. Therefore, we provide evidence that microvascular endothelial cells induce pericyte-fibroblast transition after SCI. In addition, other cells are also sources of PDGF-BB, such as macrophages, microglia, and astrocytes. Further exploration is needed to determine the effect of gene deletion of endothelial PDGF-BB on pericyte-fibroblast transition after SCI. The BSCB is destroyed after SCI, and blood-derived PDGF-BB is also released into the parenchyma of the spinal cord [34, 35]. It is unclear whether there are other sources of PDGF-BB involved in pericyte-fibroblast transition and why pericytes transition into fibroblasts other than other cells. Interestingly, the concentration of PDGF-BB in the injured core was significantly lower than that in the injured periphery. The PDGF-BB/PDGFRβ signaling pathway may not be involved in pericyte recruitment leading to abnormal angiogenesis after SCI alone.

Fibrotic scarring, BSCB leakage, the inflammatory response, and chondroitin sulfate proteoglycan deposition hinder axon regeneration after SCI [36, 37]. Although the BSCB has been shown to continue to leak after SCI [27, 38], it is unclear whether imatinib can cross the BSCB. Mathew et al. reported that imatinib orally at 250 mg/kg twice daily starting 30 min after SCI promoted functional recovery [39]. Subsequently, Kelli et al. confirmed that imatinib improved bladder function after SCI [40]. A recent study demonstrated that intraperitoneal injection of 50 mg/kg imatinib daily inhibits oxidative stress responses [41]. In this study, we used intrathecal injection of imatinib (10 mg/kg) other than gavage to block PDGF-BB/PDGFRβ signaling. During the experiment, we injected 50 mg/kg and 30 mg/kg imatinib intrathecally and found that the mice could not tolerate it, resulting in significant weight loss and sustained decline in vitality. The results showed that pharmacologically blocking PDGF-BB/PDGFRβ could promote functional recovery and axonal regeneration after SCI. Further evaluation of the formation of fibrotic scar showed that pharmacologically blocking the PDGF-BB/PDGFRβ signaling pathway inhibited the formation of fibrotic scar and the population of fibroblasts. Combined with the unique honeycomb structure formed by PDGFRβ^+^ cells at the position where the injury core is separated from the vascular wall, we speculate that imatinib inhibits the proliferation of fibroblasts and promotes the apoptosis of fibroblasts. Persistent blockade of the PDGF-BB/PDGFRβ signaling pathway may inhibit the proliferation and promote the apoptosis of fibroblasts after pericyte-fibroblast transition to form fibrotic scar. Interestingly, intrathecal injection of imatinib did not completely inhibit fibrotic scar formation. Fibrotic scarring is beneficial to rapid wound healing, while completely inhibiting fibrotic scar formation is not conducive to axonal regeneration. The transforming growth factor-β (TGF-β) signaling pathway has been confirmed to be involved in pericyte transition in the process of fibrosis at the injury site [42, 43]. Our previous study confirmed that TGFβ1 was highly expressed in microglia at 7 dpi after SCI [44]. In addition, TGF-β1 is also expressed in astrocytes, macrophages, and microvascular endothelial cells after SCI [45, 46]. The PDGF-BB/PDGFRβ signaling pathway may combine with TGF-β1 signaling pathway to induce pericyte transition.

Pericyte coverage is an important sign by which microvessels maintain normal function. In a variety of brain injuries and brain degenerative diseases, loss of pericytes can cause leakage of the blood-brain barrier [47–49]. A key change in the molecular composition of the extracellular microenvironment after SCI is that the plasma protein fibrinogen passes through the leaking BSCB. The neuropathological effects of fibrinogen include axonal injury, neurite outgrowth inhibition, inhibition of remyelination, and promotion of astroglia scar formation [50]. As a mediator of inflammation, fibrinogen can activate microglia and recruit blood-borne macrophages to enter the central nervous system [51]. The inflammatory response is promoted through the CD11b/CD18 receptor on microglia and macrophage binding of fibrinogen [52]. Therefore, the inflammatory response and microvessel leakage have a mutual promotion relationship. In this study, blocking the PDGF-BB/PDGFRβ signaling pathway did not change the coverage of pericytes on microvessels, which was different from a previous report [39]. We speculate that this discrepancy may be due to the dose and the method of imatinib administration. However, leakage at the injured site was significantly decreased. The attenuation of deposited fibrinogen led to a reduction in the inflammatory response, and the reduction in the inflammatory response may rescue the leakage of fibrinogen.

## Conclusions

In summary, the present study provides evidence of pericyte-fibroblast transition in the microenvironment of the spinal cord and confirms the role of microvascular endothelial cells in inducing transition. Targeting the PDGF-BB/PDGFRβ signaling pathway by imatinib is an effective therapeutic approach for axon regeneration, relieving inflammation, and functional recovery after SCI.

## Supplementary Information

**Additional file 1: Supplementary Figure 1.** Pericytes acquire a fibroblast phenotype after SCI. **a, b** Representative immunofluorescence images taken in the spinal cords of uninjured mice and injured mice at 3, 7, 14, and 28 days post-injury (dpi) showing that PDGFRβ^+^ pericytes (red) robustly express the fibroblast markers FSP1 (green, **a**) and vimentin (green, **b**) after SCI. The nuclei are stained with DAPI (blue). The arrowheads represent PDGFRβ^+^ pericytes or fibroblasts colocalized with FSP1 (**a**) and vimentin (**b**). n = 4 mice per time point. Scale bars: 20 μm (**a** and **b**). All images are from sagittal sections. **Supplementary Figure 2.** Imatinib inhibits proliferation and promotes apoptosis of PDGFRβ^+^ cells after fibrotic scar formation. **a** and **b** Typical immunofluorescence images of PDGFRβ (red), Ki-67 (green, in **a**), cleaved caspase 3 (C-Cas3, green in **b**), and DAPI (blue) in mice treated with intrathecal injection of imatinib and PBS (control) at 14 days post-injury (dpi). Scale bars: 20 μm. All images are from sagittal sections. **c**, **d** Quantification of the percentage of proliferating PDGFRβ^+^ cells (**c**) and apoptotic PDGFRβ^+^ cells (**d**) to PDGFRβ^+^ cells. Data are expressed as mean ± s.e.m. n = 5-6 animals per group. ***p < 0.001 versus control by unpaired two-tailed Student’s t test in **c** and **d. Supplementary Figure 3.** Fibrinogen does not leak from microvessels in the normal spinal cord. Representative immunofluorescence images of CD31 (red) and fibrinogen (green) in the spinal cord of sham mice. Spinal cord tissues were obtained by infusion of PBS and 4% paraformaldehyde. The right panel shows a high magnification image of the dotted box in the left panel. All images are from sagittal sections. Scale bars: 100 μm (left panel) and 20 μm (right panel). n = 3 mice.

## Data Availability

The data are available from the corresponding authors upon reasonable request.

## Abbreviations

SCI: Spinal cord injury
BSCB: Blood-spinal cord barrier
PDGF: Platelet-derived growth factor
PDGFRβ: Platelet-derived growth factor receptor β
FSP1: Fibroblast-specific protein 1
PBS: Phosphate-buffered saline
PFA: Paraformaldehyde
FBS: Fetal bovine serum
siRNA: Small interfering RNA
NF-H: Neurofilament-heavy polypeptide
GAP43: Growth-associated protein 43
DAPI: 4′,6-Diamidino-2-phenylindole, dilactate
C-Cas3: Cleaved caspase-3
EC-CM: Endothelial cell-conditioned medium
CM: Conditioned medium
Mye-CM: Myelin-induced conditioned medium
BMS: Basso mouse scale
Dpi: Days post-injury
ANOVA: Analysis of variance
TGF-β: Transforming growth factor-β
