# Supplementary figures and images for: Imatinib inhibits pericyte-fibroblast transition and inflammation and promotes axon regeneration by blocking the PDGF-BB/PDGFRβ pathway in spinal cord injury
Source: Inflamm Regen. 2022 Sep 26;42:44. doi: 10.1186/s41232-022-00223-9 (PMC9511779; doi:10.1186/s41232-022-00223-9)

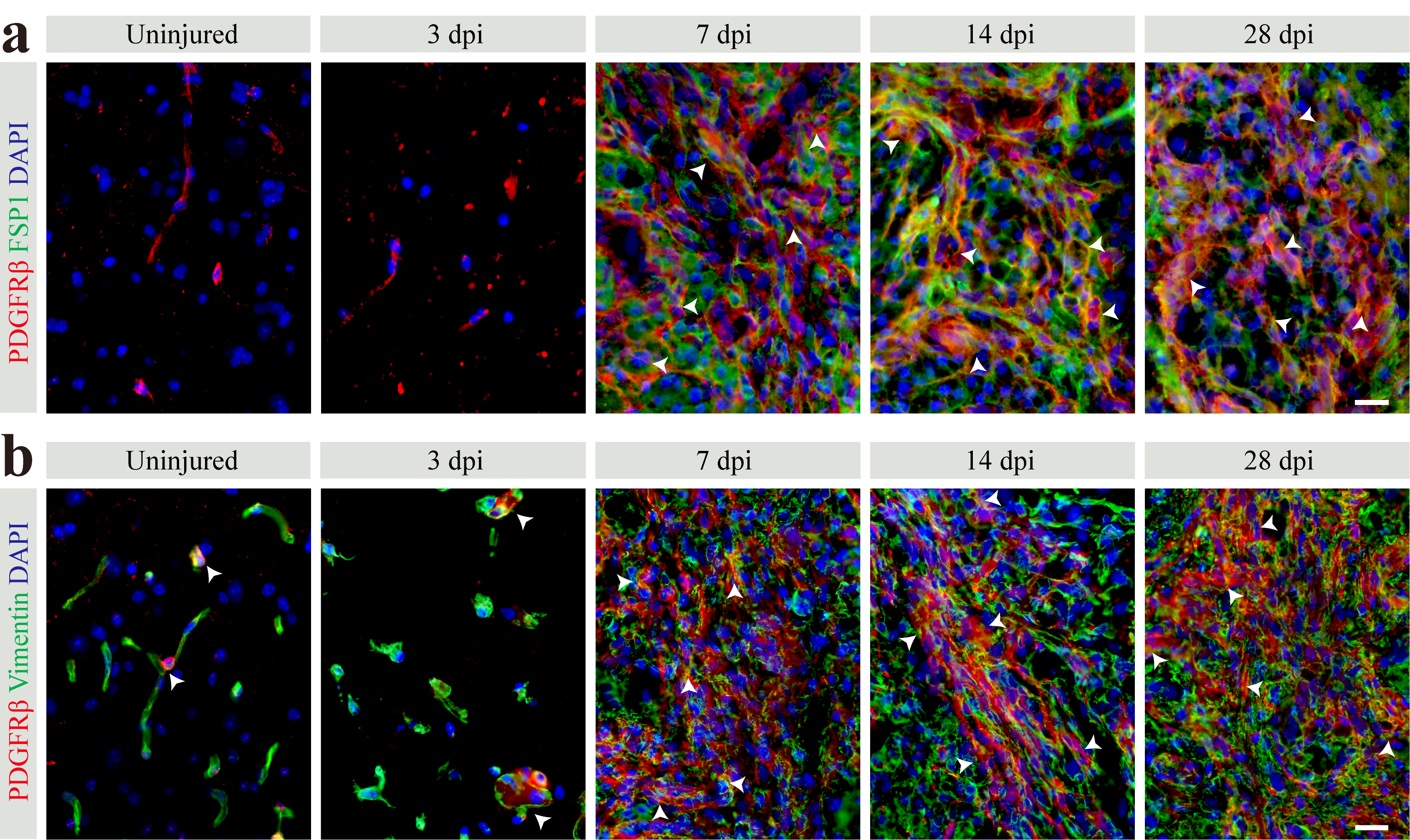

Supplement: Supplementary file 1 — Additional file 1: Supplementary Figure 1. Pericytes acquire a fibroblast phenotype after SCI. a, b Representative immunofluorescence images taken in the spinal cords of uninjured mice and injured mice at 3, 7, 14, and 28 days post-injury (dpi) showing that PDGFRβ+ pericytes (red) robustly express the fibroblast markers FSP1 (green, a) and vimentin (green, b) after SCI. The nuclei are stained with DAPI (blue). The arrowheads represent PDGFRβ+ pericytes or fibroblasts colocalized with FSP1 (a) and vimentin (b). n = 4 mice per time point. Scale bars: 20 μm (a and b). All images are from sagittal sections. Supplementary Figure 2. Imatinib inhibits proliferation and promotes apoptosis of PDGFRβ+ cells after fibrotic scar formation. a and b Typical immunofluorescence images of PDGFRβ (red), Ki-67 (green, in a), cleaved caspase 3 (C-Cas3, green in b), and DAPI (blue) in mice treated with intrathecal injection of imatinib and PBS (control) at 14 days post-injury (dpi). Scale bars: 20 μm. All images are from sagittal sections. c, d Quantification of the percentage of proliferating PDGFRβ+ cells (c) and apoptotic PDGFRβ+ cells (d) to PDGFRβ+ cells. Data are expressed as mean ± s.e.m. n = 5-6 animals per group. ***p < 0.001 versus control by unpaired two-tailed Student’s t test in c and d. Supplementary Figure 3. Fibrinogen does not leak from microvessels in the normal spinal cord. Representative immunofluorescence images of CD31 (red) and fibrinogen (green) in the spinal cord of sham mice. Spinal cord tissues were obtained by infusion of PBS and 4% paraformaldehyde. The right panel shows a high magnification image of the dotted box in the left panel. All images are from sagittal sections. Scale bars: 100 μm (left panel) and 20 μm (right panel). n = 3 mice. [file 41232_2022_223_MOESM1_ESM.zip › Supplementary Fig. 1.tif]

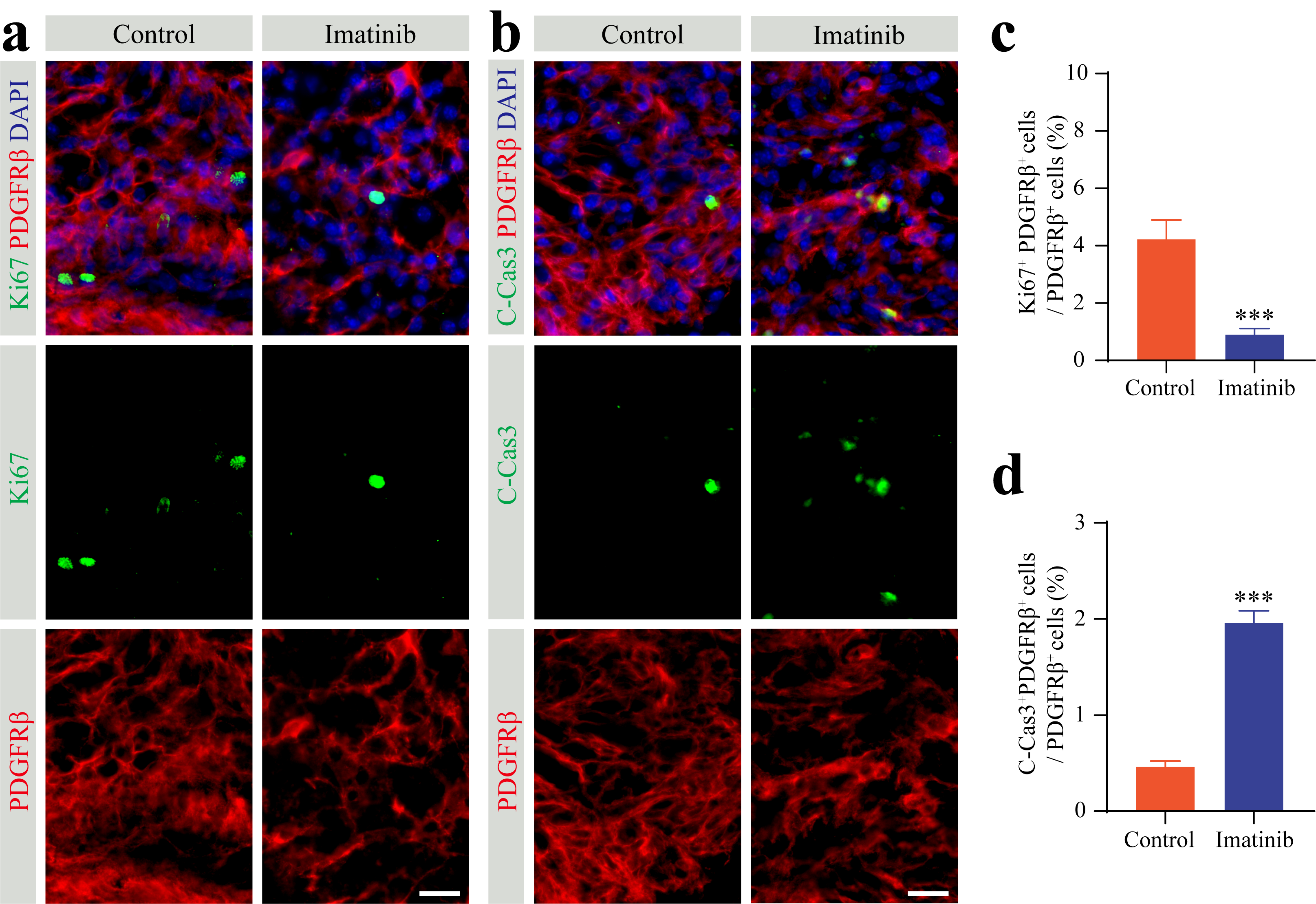

Supplement: Supplementary file 1 — Additional file 1: Supplementary Figure 1. Pericytes acquire a fibroblast phenotype after SCI. a, b Representative immunofluorescence images taken in the spinal cords of uninjured mice and injured mice at 3, 7, 14, and 28 days post-injury (dpi) showing that PDGFRβ+ pericytes (red) robustly express the fibroblast markers FSP1 (green, a) and vimentin (green, b) after SCI. The nuclei are stained with DAPI (blue). The arrowheads represent PDGFRβ+ pericytes or fibroblasts colocalized with FSP1 (a) and vimentin (b). n = 4 mice per time point. Scale bars: 20 μm (a and b). All images are from sagittal sections. Supplementary Figure 2. Imatinib inhibits proliferation and promotes apoptosis of PDGFRβ+ cells after fibrotic scar formation. a and b Typical immunofluorescence images of PDGFRβ (red), Ki-67 (green, in a), cleaved caspase 3 (C-Cas3, green in b), and DAPI (blue) in mice treated with intrathecal injection of imatinib and PBS (control) at 14 days post-injury (dpi). Scale bars: 20 μm. All images are from sagittal sections. c, d Quantification of the percentage of proliferating PDGFRβ+ cells (c) and apoptotic PDGFRβ+ cells (d) to PDGFRβ+ cells. Data are expressed as mean ± s.e.m. n = 5-6 animals per group. ***p < 0.001 versus control by unpaired two-tailed Student’s t test in c and d. Supplementary Figure 3. Fibrinogen does not leak from microvessels in the normal spinal cord. Representative immunofluorescence images of CD31 (red) and fibrinogen (green) in the spinal cord of sham mice. Spinal cord tissues were obtained by infusion of PBS and 4% paraformaldehyde. The right panel shows a high magnification image of the dotted box in the left panel. All images are from sagittal sections. Scale bars: 100 μm (left panel) and 20 μm (right panel). n = 3 mice. [file 41232_2022_223_MOESM1_ESM.zip › Supplementary Fig. 2.tif]

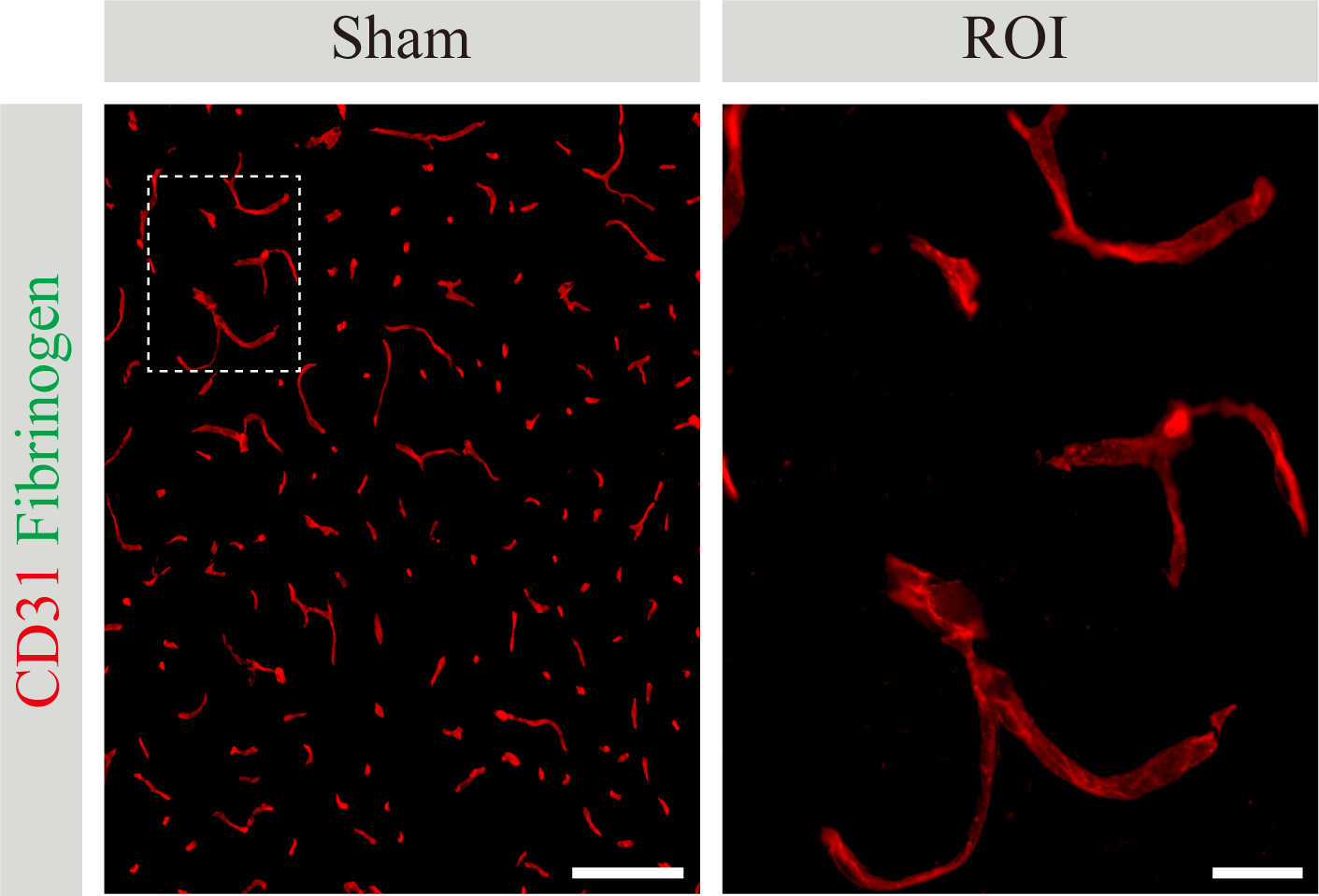

Supplement: Supplementary file 1 — Additional file 1: Supplementary Figure 1. Pericytes acquire a fibroblast phenotype after SCI. a, b Representative immunofluorescence images taken in the spinal cords of uninjured mice and injured mice at 3, 7, 14, and 28 days post-injury (dpi) showing that PDGFRβ+ pericytes (red) robustly express the fibroblast markers FSP1 (green, a) and vimentin (green, b) after SCI. The nuclei are stained with DAPI (blue). The arrowheads represent PDGFRβ+ pericytes or fibroblasts colocalized with FSP1 (a) and vimentin (b). n = 4 mice per time point. Scale bars: 20 μm (a and b). All images are from sagittal sections. Supplementary Figure 2. Imatinib inhibits proliferation and promotes apoptosis of PDGFRβ+ cells after fibrotic scar formation. a and b Typical immunofluorescence images of PDGFRβ (red), Ki-67 (green, in a), cleaved caspase 3 (C-Cas3, green in b), and DAPI (blue) in mice treated with intrathecal injection of imatinib and PBS (control) at 14 days post-injury (dpi). Scale bars: 20 μm. All images are from sagittal sections. c, d Quantification of the percentage of proliferating PDGFRβ+ cells (c) and apoptotic PDGFRβ+ cells (d) to PDGFRβ+ cells. Data are expressed as mean ± s.e.m. n = 5-6 animals per group. ***p < 0.001 versus control by unpaired two-tailed Student’s t test in c and d. Supplementary Figure 3. Fibrinogen does not leak from microvessels in the normal spinal cord. Representative immunofluorescence images of CD31 (red) and fibrinogen (green) in the spinal cord of sham mice. Spinal cord tissues were obtained by infusion of PBS and 4% paraformaldehyde. The right panel shows a high magnification image of the dotted box in the left panel. All images are from sagittal sections. Scale bars: 100 μm (left panel) and 20 μm (right panel). n = 3 mice. [file 41232_2022_223_MOESM1_ESM.zip › Supplementary Fig. 3.tif]
